# ^10^Boron Is Mobile in Cowpea Plants

**DOI:** 10.3389/fpls.2021.717219

**Published:** 2021-10-15

**Authors:** Sylvia Leticia Oliveira Silva, Renato de Mello Prado, Cassio Hamilton Abreu-Junior, Gilmara Pereira da Silva, Gabriel Barbosa da Silva Júnior, José Lucas Farias da Silva

**Affiliations:** ^1^Department of Agronomy, Federal Institute of Maranhão, Pinheiro, Brazil; ^2^Department of Agricultural Production Sciences, Soils and Fertilizers Sector, São Paulo State University (UNESP), Jaboticabal, Brazil; ^3^Center for Nuclear Energy in Agriculture, Universidade de São Paulo (USP), Piracicaba, Brazil; ^4^Soil Department, State University of Mato Grosso (UNEMAT), Alta Floresta, Brazil; ^5^Department of Plant Science, Research Center of Agricultural Sciences, Federal University of Piauí, Teresina, Brazil

**Keywords:** *Vigna unguiculata* (L.) Walp, mobility, marked micronutrient, boron fertilization, foliar spraying, phloem

## Abstract

Cowpea [*Vigna unguiculata* (L.) Walp] is cultivated in tropical and subtropical regions worldwide, but its production is usually limited by boron (B) deficiency, which can be mitigated by applying B *via* foliar spraying. In plants with nutrient mobility, the residual effect of foliar fertilization increases, which might improve its efficiency. An experiment was carried out to evaluate the concentration and mobility of the B isotopic tracer (^10^B) in different organs of cowpea plants, after the application of this micronutrient in the growing media and also to leaves. Treatments were designed based on B fertilization as follows: without B in the growth media, with ^10^B applied *via* foliar spraying (^10^B-L), with B in the growth media (substrate) and ^10^B *via* foliar spraying (^10^B-L + B-S), and with ^10^B in the growth media (substrate) without foliar spraying (^10^B-S), and a control without fertilization. A redistribution of ^10^B was observed in new leaves when the element was supplied *via* foliar spraying, resulting in greater leaf area, dry mass and dry matter production of aerial parts, and also the whole plant. ^10^Boron was redistributed when applied *via* foliar spraying in cowpea plants, regardless of the plant's nutritional status, which in turn might increase internal B cycling.

## Introduction

Cowpea [*Vigna unguiculata* (L.) Walp] is a crop of high nutritional value because of its dietary proteins and all essential amino acids, carbohydrates, vitamins, minerals, and fibers (Devi et al., 2015). One of the most important micronutrients known to increase its productivity is boron (B) (Silva et al., 2018). B deficiency in plants is a result of the low concentration of this element in the soil and is widely disseminated in the most diverse areas of cultivation in the world (Wimmer and Eichert, 2013), distributed in South and Southeast Asia, Eastern Australia, New Zealand, Africa, North and South America, and Northern Europe (Lehto et al., 2010). Such a deficiency occurs because the available fraction of total B in the soil is considerably small (1–3%) (Brdar-Jokanović, 2020). In addition, B availability is affected by many factors including soil texture, the nature of clayey minerals, pH, organic matter content, irrigation sources, inter-relation with other elements, water deficit, light intensity, and environmental conditions such as moderate to strong rainfalls (Moraghan and Mascagni, 2018).

The deficiency of B causes biological damages in plants, with special regard to disturbances in the formation of cell walls (Chormova et al., 2014). In addition, meristematic growth can be negatively affected, with characteristic symptoms appearing in new leaves and causing deformities in the foliar limb, as observed in species with restricted mobility of B, such as wheat (*Triticum sativum* L.) and barley (*Hordeum vulgare* L.) (Wimmer and Eichert, 2013). However, symptoms of B deficiency can also occur in old leaves when the nutrient is mobile in the phloem of the plant. In plants that have B mobility, a high cycling capacity of B is observed, which increases its nutritional efficiency and reduces the risks associated with B deficiency. It is possible to infer that plants with mobile B have this strategy to increase their tolerance to B shortages (Wang et al., 2015), but most plant species had a low mobility of B (Brdar-Jokanović, 2020).

A high mobility of B has been reported for some plant species that contain polyols (sorbitol, mannitol, and dulcitol) in their phloem, due to the complexation of B by cis-diol groups that facilitate the transport of nutrients in the plant (Bieleski and Briggs, 2005). In addition, B is associated with the transport of solutes (sugars) (Gauch and Dugger, 1953; Bellaloui et al., 1999). The concentration of different polyols is variable among species (Bieleski, 1982), and through transgene processes, the introduction of a gene to increment the production of sorbitol is made possible, which consequently confers mobility to B, as reported for tobacco plants (*Nicotiana tabacum* L.) (Brown et al., 1999). Therefore, in plants containing high concentrations of polyols in their phloem, B can be considered mobile, like species of the Fabaceae family, such as soybean (*Glycine max* L.) (Will et al., 2011), white lupin (*Lupinus albus* L.) (Huang et al., 2008), peanut (*Arachis hypogaea* L.) (Konsaeng et al., 2010), and in members of other families, such as olive (*Olea europaea*) (Hegazi et al., 2018) and citrange (*Citrus sinensis* L.) (Wu et al., 2019). Cowpea belongs to the Fabaceae family, and thus, it is possible to infer that the phloem mobility of B might occur in this species; however, no studies were performed with this species to date.

The mobility of B can also be affected by the plant nutritional status, considering that in plants cultivated under B deficiency, the mobility of this element is impaired in comparison with plants grown under B sufficiency, as reported by Konsaeng et al. (2010) and Will et al. (2011). These authors reported that plants cultivated under B deficiency have a limited mobility due to a rapid complexation of B into stable compounds in the cell wall, which reduces its availability for translocation in the organism; however, this might not be true for other species of plants. If the mobility of B depends on the potential of the species in producing polyols and non-alcohol sugars in the phloem, which may occur in plants under distinct nutritional states, it can be presumed that this is not an important factor to alter B mobility in the plant.

The use of isotopic techniques is considered an alternative method to identify B mobility in plants, which is a precise analytical tool in this kind of evaluation. This is because B has two stable isotopes with atomic masses of 10 and 11 that have the same biological functions in the organism and are used as a tracer in plants (Geilert et al., 2019), whether perennial like citrus (Du et al., 2020) or annual like cotton (*Gossypium hirsutum* L.) (Bogiani et al., 2014).

Understanding B mobility in plants has relevant agricultural implications, especially regarding the proper correction of the deficiency of this micronutrient with fertilization. When B mobility is low in the phloem, its foliar supply will not provide its distribution and thus will not meet the nutritional demands of the organs of the plant. In this condition, in order to obtain an efficient fertilization with constant nutrition of new leaves, B must be supplied in the root system and then transported by the xylem (Prado, 2021). However, a mobile micronutrient absorbed by leaves after several days of foliar spraying can be translocated to newly emerged leaves, ensuring the nutrition of deficient leaves and consequently resulting in higher productivity. Thus, this internal cycling of B can improve the effect of foliar spraying on the plant.

Based on previous findings, the following hypotheses were raised: (i) ^10^B applied *via* foliar sprays is mobile in cowpea plants; and (ii) the nutritional status of plants regarding B will not alter its internal mobility. To test these hypotheses, an experiment was conducted with the aim of evaluating whether ^10^B applied *via* foliar spraying can be redistributed to other parts of cowpea plants regardless of their nutritional status. In case these hypotheses are accepted, it will be possible to safely indicate foliar spraying of B, aiming to meet the nutritional demands of plants for this micronutrient, which would imply a change in the management of this nutrient to gain efficiency and to increase the sustainability of cowpea crops in several regions of the world that are deficient in this nutrient.

## Materials and Methods

This study was carried out inside a greenhouse at the São Paulo State University (UNESP), Campus of Jaboticabal/SP, Brazil. Polypropylene pots (3 dm^3^) filled with washed sand of medium texture were used in this experiment as growth media. Cowpea seedlings of the cultivar “caupi BRS-Guariba” were transplanted to the pots 13 days after sowing, when the plants had two or three pairs of completely formed leaves.

Plants were irrigated on a daily basis using a Hoagland and Arnon (1950) solution without B. The source of iron (Fe) was Fe-EDDHMA. The pH of the solution was adjusted to the range of 5.5–6.0, as recommended by Freire Filho et al. (2005). The nutrient solution was diluted to 25% of the ionic strength during the first week of cultivation, 50% during the second week, and 100% from the third week until the end of the experiment. A polypropylene collector was placed at the base of each pot, allowing drainage and retention of the excessive nutrient solution. At the end of the day, the solution was manually restored in the growth media.

**Figure 1:**
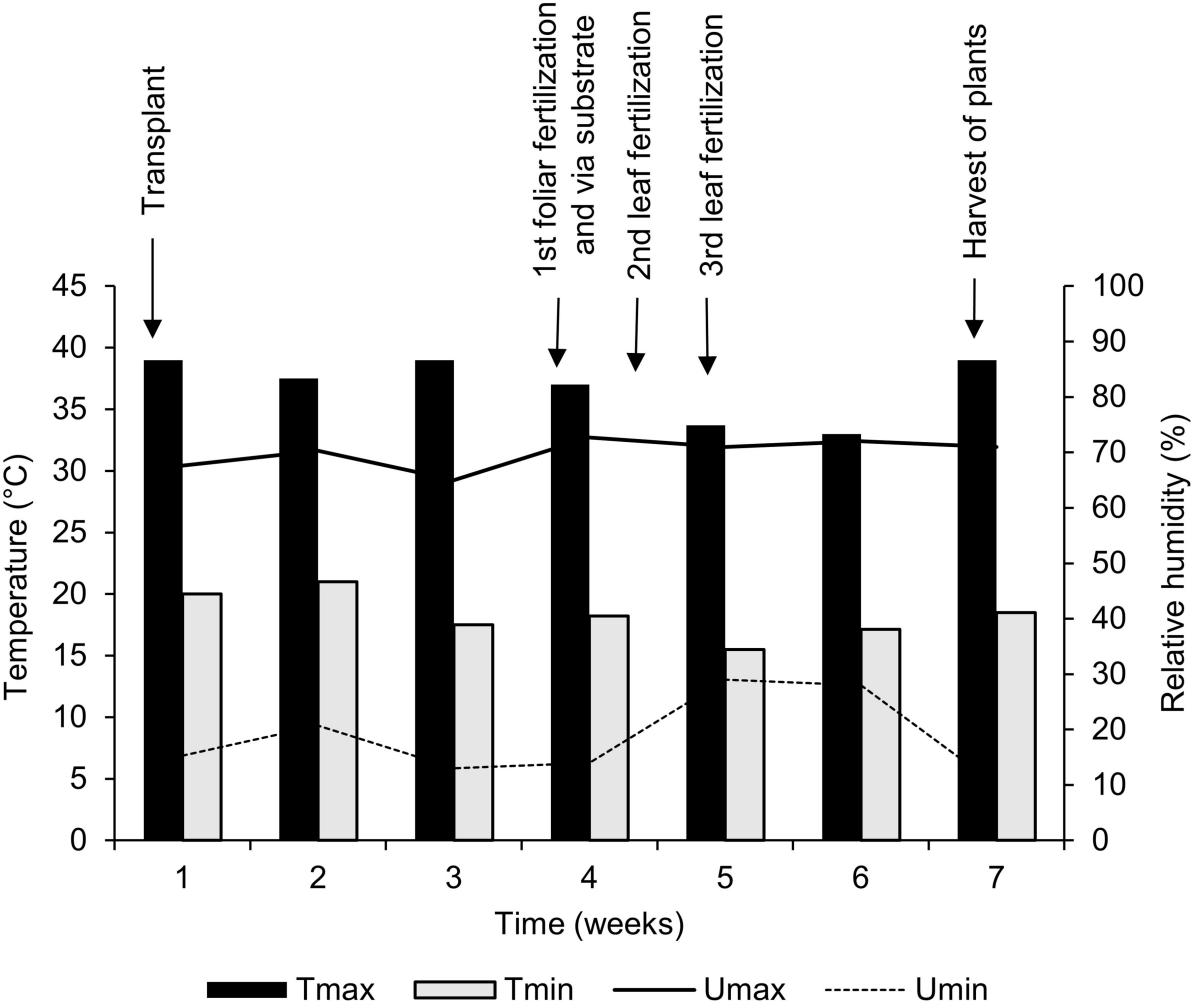
Maximum (*T*_max_) and minimum (*T*_min_) air temperature, and maximum (*U*_max_) and minimum (*U*_min_) relative humidity inside the greenhouse throughout the experimental period.

Data on air temperature and relative moisture inside the greenhouse were daily registered, with the aid of a digital thermometer (ITH-2250; Instrutemp^®^, São Paulo/SP, Brazil), from the transplantation period until the harvest of the plants, throughout 7 weeks of cultivation (Figure 1).

The experiment consisted of four treatments: the first treatment had no B application in the growth media but ^10^B was applied *via* foliar spraying (^10^B-L); in the second treatment, plants were fertilized with B in the growth media and ^10^B *via* foliar spraying (^10^B-L + B-S); and in the third treatment, fertilization with ^10^B was performed in the growth media without foliar spraying (^10^B-S). In addition, a control treatment was designed without B fertilization in both leaves and growth media. Treatments were arranged in randomized complete blocks with five replicates.

In relation to the foliar fertilization, newly formed leaves that were completely expanded received 1.4 ml (4.76 mg of B per plant) of a solution containing B at a concentration of 3.4 g L^−1^ (Silva et al., 2018) at 25, 30, and 35 days after transplanting (DAT). The applications of B were carried out at 17 h, with the use of a 5-cm plastic rod with cotton fixed in its tip, enabling the distribution of the solution on the leaf surface. The volume of the applied solution was measured for each plant, considering the weights of the rod embedded with the solution (before foliar application) and after the application.

Before the foliar application of ^10^B, the culture medium was protected with cotton and plastic bags, so that the foliar solution provided would not reach the medium, ensuring the exclusive absorption of ^10^B by the leaves. The leaves that received B directly by spraying had their leaflets marked with cotton thread immediately after the application of the treatments.

Boric acid (17% B) was used as source for treatments ^10^B-L + B-S and ^10^B-S, with the latter enriched with ^10^B atoms. Applications were performed once at 25 DAT (fourth week after transplantation). In both treatments, a solution containing B at a concentration of 46 μmol L^−1^ was used, with 100 ml being applied in each pot.

At the 45th DAT, with the emergence of new leaves after the applications, the plants were harvested and separated into leaves that received ^10^B and leaves emitted after the application of ^10^B. To determine the leaf area (cm^2^), a portable digital meter (L-31000C Model) (Li-COR INC, Lincoln, NE, USA) was used. Then, the leaves were washed in distilled water. All leaves were sequentially washed with a cotton embedded in a detergent solution (1 ml L^−1^), followed by distilled water, by a solution of HCl (24.9 ml L^−1^), and again with distilled water, in order to eliminate the micronutrients that were not absorbed and remained on the surface of the plant, according to the recommendations made by Boaretto et al. (2004). The harvested plant material was separated into roots, leaves, and stems. Samples were dried in a forced-ventilation oven at 65°C until constant weight, to evaluate the dry matter contents of the different parts of the plants.

The concentration of total B was determined by the method of azomethine-H in an extract obtained by dry digestion (Tedesco et al., 1995). In order to determine the isotopic abundance of ^10^B (% of ^10^B atoms) in samples, the extracts were submitted for an isotopic analysis of ^10^B and ^11^B using a mass spectrometer with plasma source (ICP-MS Agilent 7500ce) to the Laboratory of Plants Mineral Nutrition “Euripedes Malavolta” at the Center for Nuclear Energy in Agriculture, Universidade de São Paulo.

The percentage of ^10^B in the part coming from the fertilizer (%^10^B_ppf_) was calculated for the different parts of the plant based on Equation 1:


$$\begin{array}{l} {\%}^{10}{\text{B}}_{\text{ppf}}=[({\%}^{10}\text{B}\;\text{sample}-{\%}^{10}\text{B}\;\text{natural})/({\%}^{10}\text{B}\;\text{fertilizer}\\ -{\%}^{10}\text{B}\;\text{natural})]*\;100 \end{array}$$

The natural abundance of ^10^B in plant samples was 19.85%, while the abundance of ^10^B in the fertilizer was 99.0%.

In order to calculate the concentration of ^10^B in the leaves emitted after the application of ^10^B from the fertilizer, Equation 2 was used:


$$\begin{array}{l} {\text{B}}_{\text{ppf}}\;\left(\text{mg}{\text{kg}}^{-1}\right)=\left(\%{\text{B}}_{\text{ppf}}\;*mg{\text{kg}}^{-1}B\right)/100. \end{array}$$

The data were verified for normality (Shapiro–Wilk test) and homogeneity of variances (Levene's test) and were submitted to a variance analysis by the *F* test (*p* < 0.05); the mean values of treatments were compared by Tukey's test (*p* < 0.05) using the statistical software SAS^®^ (Cary, NC, USA).

## Results

### Concentration and Accumulation of B and Growth of Cowpea Plants

**Figure 2:**
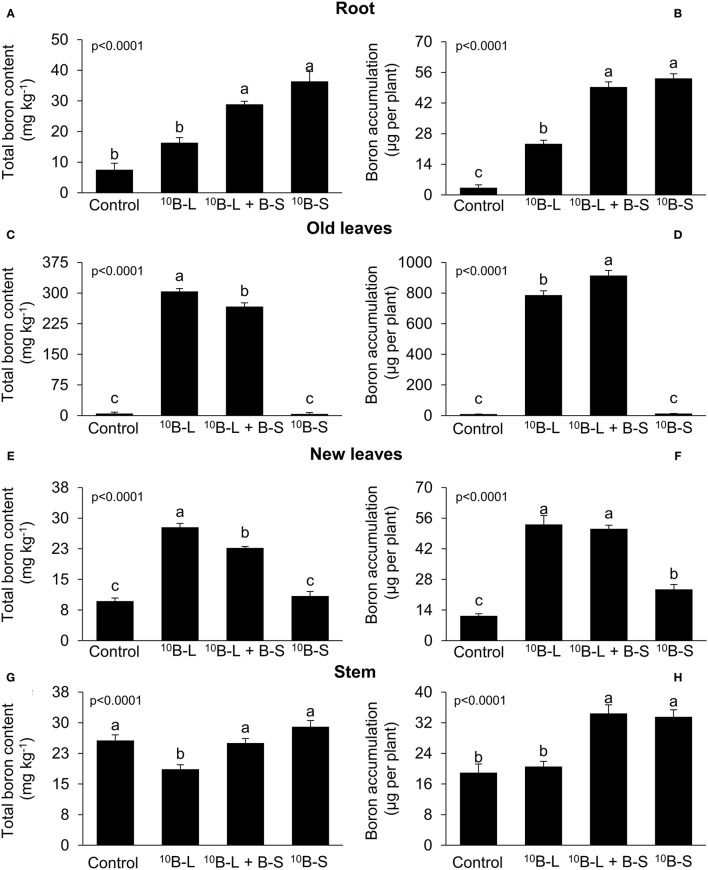
Boron concentration and accumulation on the roots **(A,B)**, old leaves (pulverized part) **(C,D)**, new leaves **(E,F)**, and stems **(G,H)** in the function of the application of marked (^10^B) and unmarked B forms of the element. Control, no B fertilization in the leaf/the foliage and substrate (growth media); ^10^B-L, without B on the substrate (growth media) and with ^10^B *via* leaf; ^10^B-L + B-S, fertilization with B on the substrate (growth media) and ^10^B *via* the leaf/the foliage; ^10^B-S, fertilization with ^10^B on the substrate (growth media) and without fertilization with B *via* leaf. Bars represent the standard errors of means. Lowercase letters indicate significant differences among treatments, according to Tukey's test at a 5% probability level. Values are presented as mean ± SE.

The application of ^10^B-S and ^10^B-L + BS resulted in an increase of 284% and 384%, respectively, in the contents of B and the accumulation of the micronutrient in the roots of cowpea plants (Figures 2A,B) compared with the control. Fertilization with ^10^B-L and ^10^B-L + B-S resulted in higher contents (5,829 and 5,106%, respectively) and accumulation (7,692 and 8,949%, respectively) of total B in old leaves (Figures 2C,D) compared with the control. The application of the nutrient *via* foliar spraying (^10^B-L) provided a higher concentration (187%) of B in new leaves (Figure 2E) compared with the control. However, the exclusive application in leaves (^10^B-L) was similar to the combined application in leaves and growth media (^10^B-L + B-S), and both promoted an increase of 370 and 352%, respectively, in the accumulation of total B in new leaves (Figure 2F) in comparison with the control.

Plants treated with ^10^B-L had higher concentrations of total B in the stems, in comparison with other treatments, even though a significantly higher accumulation of the micronutrient was found in ^10^B-L + B-S and ^10^B-S (Figures 2G,H).

**Figure 3:**
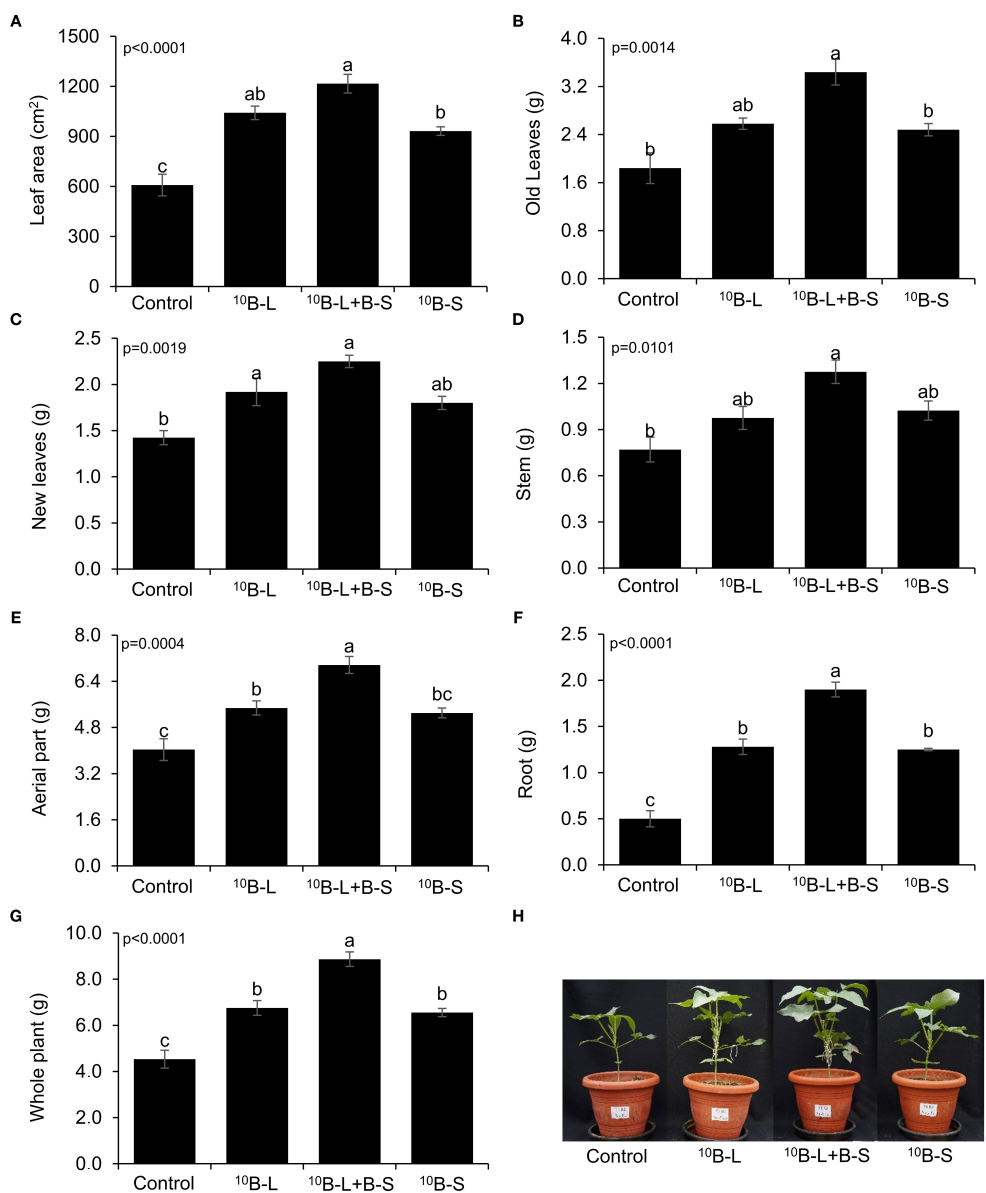
Leaf area **(A)**, old leaves **(B)**, new leaves emerged after foliar spraying **(C)**, stems **(D)**, aerial part **(E)**, roots **(F)**, and whole plant **(G)**, depending on the application of B in cowpea plants, and photographs of the plants showing the effects of treatments **(H)**. Control, without boron fertilization on the substrate (growth media) and leaves; ^10^B-L, without B on the substrate (growth media) and with ^10^B *via* leaf; ^10^B-L + B-S, fertilization with B on the substrate (growth media) and ^10^B *via* the leaf/the foliage; ^10^B-S, fertilization with ^10^B on the substrate (growth media) and without fertilization with B *via* the leaf/the foliage. Bars represent the standard errors of means. Lowercase letters indicate significant differences among treatments, according to Tukey's test at a 5% probability level. Values are presented as mean ± SE.

When ^10^B was supplied *via* foliar in treatments ^10^B-L and ^10^B-L + B-S, there was an increase in the leaf area by 71 and 99%, respectively, when compared with the control (Figure 3A). Consequently, the highest dry matter production of old leaves was observed in the ^10^B-L + B-S treatment, showing an increase of 87% compared with the control treatment (Figure 3B).

The dry mass of new leaves was increased by 35 and 58% when plants were treated with ^10^B-L and ^10^B-L + B-S, respectively, in comparison with the control (Figure 3C). However, the effect of B from the treatment ^10^B-L + B-S stood out from the other treatments regarding the production of dry matter in the aerial parts, roots, and the whole plant as well (Figures 3E,G). The effects of treatments were visually observed, demonstrating the importance of the foliar application of B, associated or not with growth media fertilization in cowpea plants (Figure 3H).

### B Mobility in Cowpea Plants

It was observed an increase in the concentration of B in the new leaves of 174 and 134%, respectively, when supplied *via* foliar spraying (^10^B-L and ^10^B-L + B-S), when compared with the control treatment, but the application in the growth media (^10^B-S) was statistically similar to the control (Table 1). However, the highest concentration of B_ppf_ in new leaves came from the provision of the nutrient *via* isolated foliar spraying or in association with B being added in the growth media (^10^B-L and ^10^B-L + B-S), presenting an increase of 664 and 478%, respectively, when compared with the supply only *via* growth media (^10^B-S).

**Table 1:** Leaf concentration of B in new leaves emerged after leaf application, percentage of ^10^B from fertilizer in cowpea plants submitted to the application of marked and unmarked B, on the substrate and on the leaves.

**Treatments**	**^**10**^B content**	** ${\text{B}}_{\text{ppf}}^{\left(1\right)}$ **
	**mg kg^**−1**^**	**mg kg^**−1**^**
Control	9.69 b	0.07 c
^10^B-L	26.60 a	16.59 a
^10^B-L + B-S	22.70 a	12.55 b
^10^B-S	10.95 b	2.17 c
*F* values	38.07^**^	95.31^**^
DMS	5.53	3.31
C.V. (%)	17.5	23.3

*C.V., coefficient of variation; DMS, significant minimum difference; ^(1)^B_ppf_, boron in the plant derived from fertilizer. Control, no B fertilization in leaves and substrate (growth media); ^10^B-L, without B on the substrate (growth media) and with ^10^B via leaf; ^10^B-L + B-S, fertilization with B on the substrate (growth media) and ^10^B via leaf; ^10^B-S, fertilization with ^10^B on the substrate (growth media) and without fertilization with B via leaf. Lower case letters differ between treatments, according to the Tukey test at 5% probability*.

** *Significant at 1%*.

**Figure 4:**
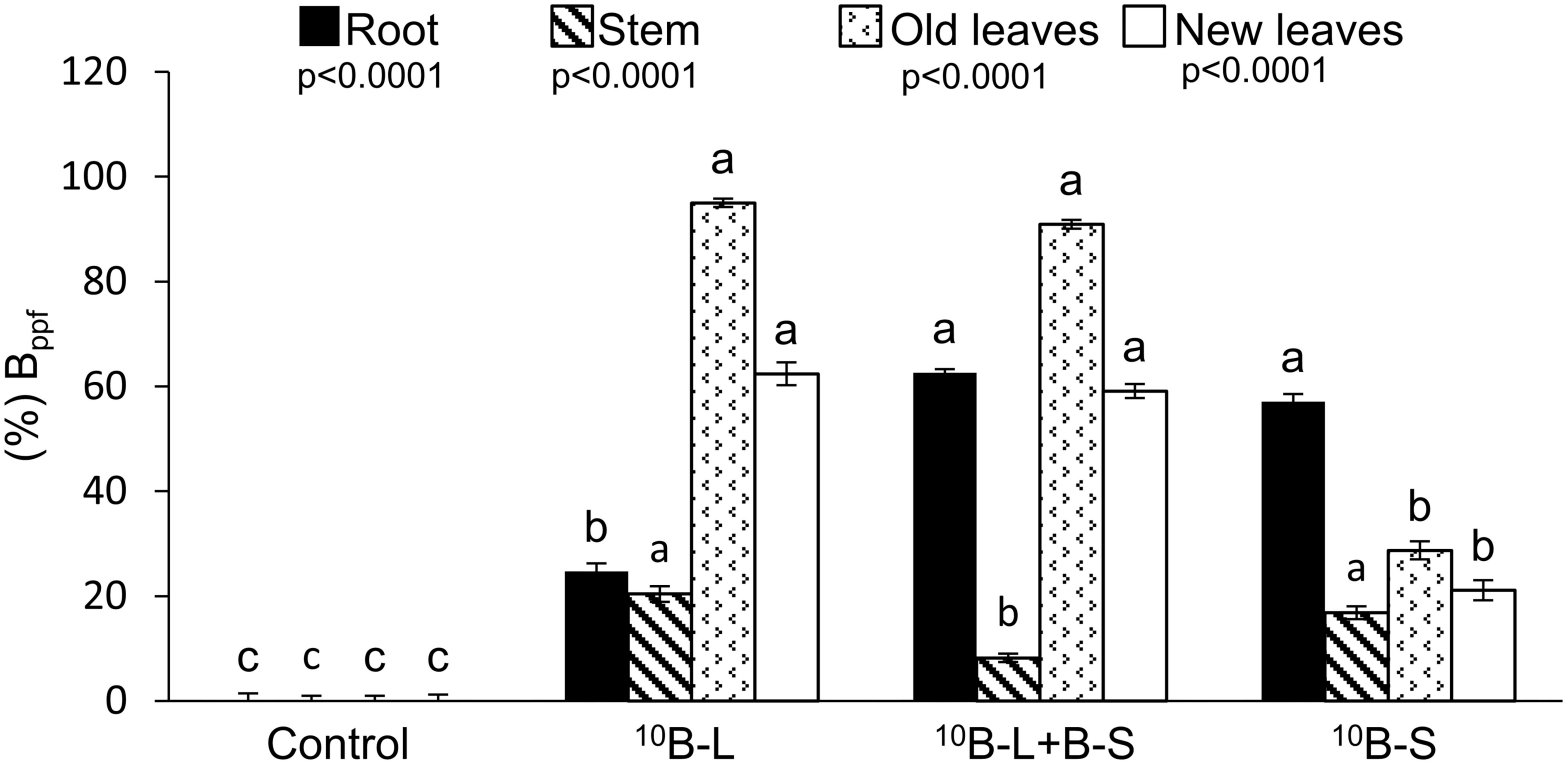
Percentage of B fertilizer in the plant (%B_ppf_) in the roots, stems, old leaves (pulverized part), and new leaves in different treatments. Control, without boron fertilization on the substrate (growth media) and leaves; ^10^B-L, without B on the substrate (growth media) and with ^10^B *via* the foliage; ^10^B-L + B-S, fertilization with B on the substrate (growth media) and ^10^B *via* the foliage; ^10^B-S, fertilization with ^10^B on the substrate (growth media) and without fertilization with B *via* the foliage. Error bars represent the standard errors of the means. Lowercase letters indicate significant differences among treatments, according to Tukey's test at a 5% probability level. Values are presented as mean ± SE.

Higher percent values of B_ppf_ were found both in old (231 and 216%) and new leaves (195 and 180%) (Figure 4) of cowpea plants, which received ^10^B-L and ^10^B-L + B-S treatments, respectively, when compared to the treatment with the supply of ^10^B only *via* growth media (^10^B-S). Fertilization with ^10^B-L and B-S isolated resulted in higher percentages of B_ppf_ in the stems (20 and 17%) (Figure 4), but treatments ^10^B-L + B-S and ^10^B-S resulted in higher B_ppf_ in the roots of the plants (63 and 57%).

## Discussion

Boron must be optimally absorbed in order to generate a significant response to its application in cowpea plants (Silva et al., 2018). In this sense, our study indicated that applying B *via* foliar spraying in association with growth media fertilization (^10^B-L + B-S) resulted in a greater accumulation of this nutrient in plant organs compared with the control treatment without B supply (Figure 2). The greatest accumulation of B provided by this treatment resulted in a higher dry mass production of aerial parts, roots, and the whole plant as well (Figure 3), indicating that this species is demanding and responsive to the application of B. Plants with high demands for B usually present high contents of this nutrient in pectins that compose the cell wall (Hu et al., 1996). Boron acts in the biosynthesis of the cell wall, assisting Ca in the deposition of pectates that will be part of these structures, in addition to composing cis-diol-borate complexes, which make up the plasmalemma (Prado, 2021). When performing its biological functions, the benefits of B for plants growth become clear, as verified in multiple crops (Prado et al., 2013; Silva et al., 2016; Maity et al., 2021).

After a few days of foliar spraying, a significant response of plants facing B fertilization can be observed by the emission of new leaves that can be nourished with B derived from the leaf that received the fertilizer. For this to occur, the nutrient would have to move significantly from the leaves that received the element *via* foliar application, in order to meet the demand of the younger parts of the plant, which have emerged after the B spraying. Aiming to discriminate that the increase of B in new leaves emerging after foliar spraying comes from the fertilizer applied in other leaves, and not from other sources, ^10^B can be used. This finding was observed in cowpea plants treated with foliar sprays of ^10^B (^10^B-L), either with or without the association of growth media fertilization (^10^B-L + B-S), resulting in a greater accumulation of B in newly emerged leaves, after fertilization in these treatments (Figure 2F).

Therefore, the foliar application of B resulted in a residual effect that benefited new leaves, corroborating the study of Wu et al. (2019) with citrange. It was also observed that these treatments increased the total B concentration of new leaves (Table 1), reaching an adequate range for this species (21 to 35 mg kg^−1^) (Silva et al., 2018).

When using this isotopic technique, it became clear that the newly emerged leaves after receiving B in different ways (^10^B-L and ^10^B-L + B-S) had a higher concentration of ^10^B, %^10^B, and ^10^Bppf (Table 1), as well as the percentage of ^10^Bppf (Figure 4), in comparison with ^10^B-S. These results demonstrated that ^10^B was redistributed to young leaves, therefore, proving the first hypothesis, as it is feasible to indicate in an unprecedented way that B is mobile in cowpea plants. This finding implies in the scientific recommendation that this nutrient should be applied by spraying in this crop.

The observation made in relation to B mobility in the phloem of cowpea plants is conditioned to the complexation with polyols and other non-alcohol sugars in cis-diol groups, forming a stable compound that facilitates the transport of nutrients in the phloem (Brown and Hu, 1996). Thus, the plants that presented a high concentration of polyols in the phloem sap would be indicative of mobility. This finding induced the production of new sources of B associated with polyols, but this did not affect the mobility and the growth of the crop (Coutinho Neto et al., 2020), as this attempt does not affect the concentration of polyols in the phloem. However, studies on the mobility of B in rice (*Oryza sativa* L.) (Bellaloui et al., 2003), white lupin (Huang et al., 2008), olive (Hegazi et al., 2018), and citrange (Wu et al., 2019) demonstrated that in these species, different concentrations of polyols were found, indicating the need for further studies of those compounds in the phloem that could affect its mobility.

**Figure 5:**
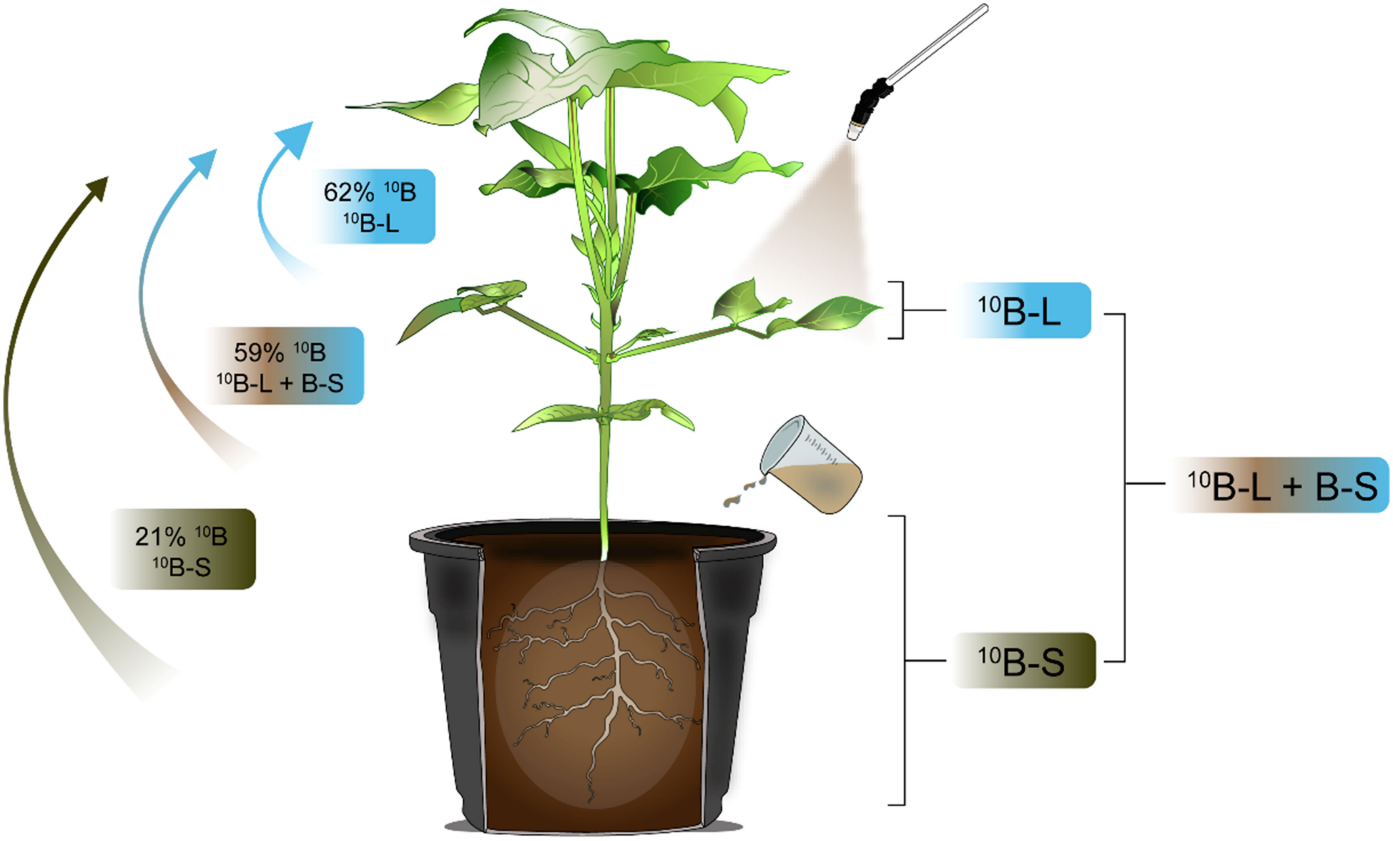
Graphical abstract of the redistribution of ^10^B applied in different ways in cowpea plants.

It is noteworthy that the intensity of B mobility applied *via* foliar spraying can be measured using the isotopic technique as shown in various studies (Konsaeng et al., 2010; Wu et al., 2019; Du et al., 2020). The exclusive foliar spraying or substrate application of B or when both fertilization techniques were conducted concomitantly, it resulted in values of ^10^Bppf equal to 62, 59, and 21%, respectively, in newly emerged leaves (Figure 5), indicating a higher mobility of B supplied *via* foliar spraying. Thus, it was verified that most of this nutrient (~60%) is mobile in this plant species, when applied to leaves. In addition, considering the same isotopic technique used for the analysis of other species of the genera Malus, Pyrus, and Prunus, ~75% of the B absorbed by leaves was redistributed to other parts of the plants and thus classified as showing B mobility in the phloem (Picchioni et al., 1995). What is important is that these levels of B mobility are sufficient to meet the demands of new leaves from older leaves that received a foliar application in a condition of B deficiency in the growth media.

In this study, it was demonstrated that the exclusive foliar application has a great agronomic importance in comparison with the root fertilization of B as both treatments resulted in a similar increment of B in the leaf area, roots, and whole plant (Figure 3). In addition, it was also observed that the accumulation of total B in the plant is smaller when the nutrient was applied in the growth media in comparison with leaf spraying (Figure 2) given the fact that B was supplied only once in the growth media during the cultivation period.

The importance of foliar spraying of B for plants growth can be explained by the optimal absorption of B and the internal cycling of this element from the leaves that received B to the leaves emerging after foliar spraying, improving their nutritional efficiency. The response of cowpea plants facing foliar application of B was also observed in other crops that present B mobility, such as peanut (Konsaeng et al., 2010), pineapple (*Ananas comosus* L.) (Siebeneichler et al., 2005), plum (*Prunus domestica* L.), cherry (*Prunus avium* L.), apple (*Malus domestica* Borkh.) (Brown and Hu, 1996), and broccoli (*Brassica oleracea* var. *italica*) (Shelp, 1988). However, for species with immobile B in the phloem, it was reported that there is a lack of response of plants to foliar spraying such as tomato (*Solanum lycopersicum* L.) (Gondim et al., 2015) and cauliflower (*Brassica olerace*a var. *botrytis*) (Alves et al., 2020). This may be due to the low residual effect of the foliar spraying of B. In other words, even though the element absorbed by the leaf met its nutritional demand, it was not redistributed to new leaves that emerged after the foliar spraying, resulting once again in deficiency symptoms. Additionally, other environmental factors might have influenced these results during foliar application, which impair crop absorption and response. In species with low mobility of B, foliar spraying is important but may need more than one application to improve crop response, depending on the level of B deficiency in the growing environment.

The treatment with only foliar spraying (^10^B-L) resulted in lower dry mass production compared with the foliar spray treatment associated with the application of B in the growing medium (^10^B-L + B-S). This might have occurred because there was only one foliar spray that could have induced B deficiency in the short term. However, the percentage of ^10^Bppf in young leaves of plants that received only the foliar spraying (^10^B-L), which presented B deficiency in the short term, was similar to the treatment that received foliar spray associated with fertilization (^10^B-L + B-S) in the growing medium. In other words, the ^10^Bppf content in new leaves that emerged after foliar spraying remained unchanged, even though the amount of B provided by the treatment (^10^B-L + B-S) was greater than the treatment (^10^B-L). This result indicates that the mobility of ^10^B in this crop is not dependent on the nutritional status of plants, seen that mobility was observed in plants under sufficiency and deficiency of B. This finding corroborates the results reported by Will et al. (2011), who indicated the occurrence of ^10^B mobility when the element was applied in soybean leaves, even under a deficiency condition of B. Therefore, the second hypothesis raised in this study can also be accepted, as it was observed that species with mobile B, such as cowpea, maintains B mobility under different nutritional statuses.

The findings presented in this study propose that the application of B in cowpea plants can be carried as in three spraying events throughout the growing cycle of the crop, being sufficient to meet its nutritional demand and to correct its deficiency, improving the sustainability of this crop. Further research should be conducted under field conditions, testing different concentrations of B in each spraying event, as well as evaluating alternative sources and its interaction with other nutrients.

## Conclusion

The B applied *via* foliar spraying in cowpea plants was redistributed to other parts of the plants, regardless of its nutritional status, demonstrating the feasibility of foliar spraying in this crop.

## Data Availability Statement

The original contributions presented in the study are included in the article/supplementary material, further inquiries can be directed to the corresponding author.

## Author Contributions

SS and JS wrote the manuscript, with contributions from all authors. RP and CA contributed to the conceptualization and administration of the project. SS, GiS, GaS and JS contributed to the research, data collection, and processing. All authors contributed to the review and improvement of the manuscript.

## Funding

This study was funded by the Coordination for the Improvement of Higher Education Personnel (CAPES), Brazil, Code 001. CA is the recipient of a research productivity fellowship from the Brazilian National Council for Scientific and Technological Development (CNPq) (grant #312728/2017-4).

## Conflict of Interest

The authors declare that the research was conducted in the absence of any commercial or financial relationships that could be construed as a potential conflict of interest.

## Publisher's Note

All claims expressed in this article are solely those of the authors and do not necessarily represent those of their affiliated organizations, or those of the publisher, the editors and the reviewers. Any product that may be evaluated in this article, or claim that may be made by its manufacturer, is not guaranteed or endorsed by the publisher.
